# Seasonal changes of faecal cortisol metabolite levels in *Gracilinanus agilis* (Didelphimorphia: Didelphidae) and its association to life histories variables and parasite loads

**DOI:** 10.1093/conphys/coy021

**Published:** 2018-07-18

**Authors:** S E Hernandez, A L S Strona, N O Leiner, G Suzán, M C Romano

**Affiliations:** 1 Departamento de Etología, Fauna Silvestre y Animales de Laboratorio, Facultad de Medicina Veterinaria y Zootecnia, Universidad Nacional Autónoma de México, Ciudad de México, CP, México; 2 Programa de Pós-Graduação em Ecologia e Conservação de Recursos Naturais, Universidade Federal de Uberlândia, MG, Brazil; 3 Laboratório de Ecologia de Mamíferos, Instituto de Biologia, Universidade Federal de Uberlândia, MG, Brazil; 4 Departamento de Fisiología, Biofísica y Neurociencias, CINVESTAV-IPN, Ciudad de México, CP, México

**Keywords:** Didelphidae, glucocorticoids, parasitism, reproduction, seasonality, semelparity

## Abstract

The aim of this study was to evaluate the role of environmental (dry versus wet season) and individual (sex, body mass and reproductive status) factors in the levels of faecal cortisol metabolites (FGCs) in *Gracilinanus agilis* faecal samples as an index of stress levels in this species; as well as its association with abundance of *Eimeria* spp, as an indicator of immunocompetence against parasites. Our study found that FGCFGCs are a reliable indicator of adrenal activity in *G. agilis*. We found that FGCFGCs increase considerably by environmental stressors like the dry season. Moreover, the observed positive association between FGCs and body mass is the result of the effect of season and reproduction in both variables. We also demonstrated that an increase in FGC levels among *G. agilis* during the dry season is associated with a rise in the probability of being infected by *Eimeria* spp. Hence, our finding supports the corticosteroid-fitness hypothesis, which predicts that increased glucocorticoids as a response to stressors usually results in decreased fitness of individuals, translated into low future survival and reproductive success, and higher parasite infection. To our knowledge, this is the first study that integrates environmental changes, hormone responses and parasite loads in a US marsupial in both empirical and experimental approaches.

## Teaser

FCMs are a reliable indicator of adrenal activity in *G. agilis*. There is an association among stress response, reproductive status and weather. Environmental stress rises in the probability of *Eimeria* spp infection in *G. agilis*

## Introduction

To cope with challenging stimuli, free-ranging animals use a variety of life-history and behavioural strategies to maintain survival and increase fitness. Among vertebrates a common physiological response to exposure to a stressor involves the activation of the hypothalamic–pituitary–adrenal axis, which increases the secretion of glucocorticoid (GC) hormones to deal with changes in allostasis (McEwen and Wingfield, 2003). Acute stress elicits a short-term increase in GCs, which mobilises energy and increases gluconeogenesis for immediate survival, while inhibiting other physiological processes (Sapolsky *et al.*, 2000). On the other hand, chronic stress usually has deleterious consequences to animals, once it leads to long-term elevation of these hormones and consequently, suppression of growth, reproduction and immune functions. Understanding the stress response of animals in the face of a challenging, changing environment is essential to their conservation (Madliger and Love, 2014).

Traditionally, the evaluation of the adrenocortical activity in response to stress in vertebrates has been achieved by determining the levels of GCs in blood. GCs can be found free in the blood plasma or bound tightly to carrier proteins in the blood. Once they exert their actions by binding to GC receptors in target tissues, they are transported to the liver where they are metabolised and then excreted as water soluble conjugates in the urine or faeces (Meikle, 1989; Brownie, 1992; Möstl and Palme, 2002). GC metabolite excretion by these last two pathways has opened the opportunity of developing techniques to assess the stress response non-invasively. In recent times, the measurement of metabolites of GCs in faecal samples has been approved as a reliable way to assess the adrenal response during stress in animals (Möstl and Palme, 2002), allowing long-term studies of the physiological response in individuals (Palme *et al.*, 2000; Romano *et al.*, 2010) as well as in populations (Dloniak *et al.*, 2004).

The stress response may vary according to sex, age and social rank of individuals (Romero, 2004). There is evidence that predator attack, increased energetic demands due to reproduction, and food shortage act as stressors, with different consequences for vertebrate survival and fitness, depending on the duration of the stress response (Martin, 2009). Due to the possible deleterious effects in their host, parasites have attracted special interest of stress response researchers. However, while some studies have found an association between parasite infection and high levels of GCs (Oppliger *et al.*, 1998; Pedersen and Greives, 2008), others have shown inconsistent results in these associations (Lafferty and Holt, 2003). Actually, the interaction between GC levels and parasites is not a simple association, as it depends on parasite virulence, host susceptibility and the synergistic effects of multiple challenging stimuli. For example, a study in white-footed mice shown that GCs levels increased in infected animals, only during the food shortage period (Pedersen and Greives, 2008). Moreover, another study found that population density has a strong effect on the association of parasite infection and GCs levels (Raouf *et al.*, 2006), with reduced GCs in response to parasite removal occurring only when the population density (e.g. colony size) is large.

Among marsupials, ultimate stress factors are reproduction, habitat loss and fragmentation, captivity and translocation, and climate change that may lead to altered food and water availability and nutritional stress (Hing *et al.*, 2014). Moreover, among several dasyurid species with short life-histories, the stress response seems to be related to stressful male–male olfactory exposure (Toftegaards *et al.*, 1999; Toftegaard *et al.*, 2002; Toftegaard and Bradley, 2003) during a short mating period coinciding with low temperatures and a reduced food supply (Bradley, 2003). The increased cortisol levels coupled to a failure in the negative GC feedback system results in impairment of immune system, high parasite infection, weight loss and organ degeneration (Naylor *et al.*, 2008), leading to post-mating mortality among males (Bradley, 2003). Along with male mortality, females usually suffer from post-reproductive senescence (Cockburn, 1997). At least, seven didelphid species present signs of male post-mating mortality, lack of generation overlap and even female disappearance after weaning their young, characterising partial or complete semelparity (Leiner *et al.*, 2008; Baladrón *et al.*, 2013; Lopes and Leiner, 2015). However, the role of stress and cortisol elevation on parasite loads and impairment of the immune system is not yet described among didelphids, as is the association between stress, cortisol and the adoption of a semelparous breeding strategy.

*Gracilinanus agilis* is a small, nocturnal didelphid that inhabits Brazilian cerrado areas. Previous study provided evidence that breeding in this species starts in July, during the dry, low food supply season (Lopes and Leiner, 2015). This species presents sexual size dimorphism, with males presenting larger body mass than females, irrespective of their age (Lopes and Leiner, 2015). Males increase their body mass and movement areas during the breeding season and disappear from the population between October and December, while females adopt a territorial strategy to defend their pups and resources and disappear from the population after weaning one to two litters in January/February, during the wet season (Lopes and Leiner, 2015). Before disappearing, males present fur loss, wounds and increased loads of *Eimeria* spp. (Apicomplexa: Eimeriidae), which is the most common gastrointestinal parasite infecting the studied *G. agilis* population (Strona *et al.*, 2015). Female infection by *Eimeria* spp. was also associated to the breeding status, with increased loads during lactation and weaning, which corresponds to the late breeding/wet season (Strona *et al.*, 2015).

The aim of this study was to evaluate the role of environmental and individual factors in the levels of cortisol metabolites in *G. agilis* faecal samples as an index of stress levels in the species. Besides the role of environmental and individual factors in the levels of cortisol, we assessed the effect of cortisol metabolite levels in *Eimeria spp*. abundance and chance of infection in *G. agilis* individuals. Hence, we tested the following hypotheses: (i) cortisol metabolites levels in faecal samples increase during the dry season when compared with the wet season, due to low food availability in the dry season, (ii) reproductive individuals present higher cortisol metabolite levels in faecal samples than non-reproductive individuals, (iii) during dry season, reproductive individuals with increased body mass present higher cortisol metabolites levels in faecal samples than leaner individuals and (iv) *G. agilis* individuals presenting increased cortisol metabolite levels in their faeces present higher parasite (*Eimeria* spp.) loads and an increased chance of being infected by *Eimeria* spp.

## Material and methods

### Study area

The study was conducted at a cerrado sensu stricto area located at the Estação Ecologica do Panga (19°09′ 20″–19° 11′10″ S and 48°23′ 20″–48^o^24′ 35″ W, MG), a small fragment of cerrado situated in Uberlândia municipality, Minas Gerais state, Brazil. The area is characterised by a tropical climate with well-defined seasons: a dry season from April to September and a wet season from October to March. Mean annual temperature is 22°C and average annual rainfall is 1650 mm, with more than 90% of the precipitation occurring the wet season (Oliveira-Filho and Ratter 2002).

### Animals

Permission to trap and handle Didelphidae was issued by SISBIO/ICMBio (Brazil) to N.L. (Permit Number: 22629-1) and all trapping and handling of didelphids agreed with the ethical principles on animal research as regulations of National Advice of Control and Animal Experimentation (CONCEA/Brazil). The protocol was approved by the Ethics Committee on Use of Animals of the Federal University of Uberlândia, Brazil (permit number: 152/13).

### Sample collection

*Gracilinanus agilis* individuals were captured in monthly sessions of four consecutive nights from April 2013 to April 2015. Live traps (Sherman model, 25 × 8 × 9 cm) were set in a capture grid containing eight parallel transects equidistant 20 m, with eight trapping stations each also separated by 20 m. In each trapping station, we placed two live traps, one in the ground and other in the understory, baited with a mixture of banana, oatmeal, peanut butter and bacon. All traps were checked in the morning (between 8 a.m. and 9 a.m.) and re-baited if necessary. Captured individuals were marked with numbered ear-tags, and then released at the point of capture. We also recorded their sex, body mass, reproductive status and point of capture. Reproductive status was evaluated based on external characters, such as perforated vagina and presence of swollen nipples or milk production as indicators of female reproduction. On the other hand, position and size of testis were used as indicators of male reproductive activity; thus, males presenting scrotal testis (>10 × 13 mm^2^) were considered reproductively active.

Since these animals normally defecate in response of acute stress, we collect all fresh faecal samples found in the trap. Nocturnal activity of *G. agilis*, starting 2 h after sunset and ending 2 h before sunrise in Central Brazil (Vieira *et al.*, 2017) ensures that each individual spent ~12–14 h in the traps. Hence, the average amount of time the study animals were in the trap before the faecal samples were collected was always <24 h. A total of 205 faecal samples, belonging to 92 individuals (61 males and 31 females) were collected. Faecal samples from the captured animals were placed in sealed containers with ice and kept at a temperature below −4°C for transport to the laboratory. In each capture session, samples from the same individuals were separated in duplicates or triplicates for parasite analysis and for hormone extraction and analysis. Samples used for hormone extraction were oven-dried at 70°C for 2 h, once in the laboratory. The dried samples were then crushed in a mortar and cleaned of food residues and stored in a new sealed container and kept in a freezer at a temperature of −20°C until they were analysed in the laboratory. On the other hand, samples used for parasite analysis were kept in a freezer at a temperature of −4°C until they were analysed in the laboratory.

### Capture stress challenge experiment

A capture stress challenge in the field was developed to determine if the levels of metabolite of cortisol in faecal samples can be attributed to physiological stress in *G. agilis*. A total of four adult *G. agilis* (two males and two females) were caught at Reserva Ecologica do IBGE (15°56′ 41″ S e 47°53′ 07″ W), situated near Brasilia (DF-Brazil), during the wet season. Each experiment was carried out over a period of 48 h during different days. The animals were captured by live traps (model Sherman 25 × 8 × 9 cm) with coupled clocks to register the capture time. Traps were set during the afternoon, using bait marked with food colourant, and after 12 h traps were checked. Any fresh faecal sample without colourant found in the trap was collected in a sealed container and kept in a temperature below −4°C for transport to the laboratory. This first faecal sample was categorised as time 0. The opossums were then placed in a cloth bag and transported 2 km to a field laboratory (~15 min by car), and placed in containment cages for small mammals (size width 30, high 20, deep 13 cm) with water and food *ad libitum*. The cages were maintained covered with a cloth at a room temperature 25°C and humidity 60%. Once the animals were placed in the cages at the field laboratory, pooled coloured faecal samples were collected at different times to assess cortisol levels after capture. The first one was 24 h after placed in cage, and the second one 48 h after capture. At the end of this period, the animals were released back into the specific capture location.

### Hormone extraction and analysis

The extraction of GCs in faecal samples was based on the protocol developed by Graham and Brown (1996).

Diet has an important effect in the levels of hormone metabolites found in faecal samples. There is evidence that fibre and water in the diet affect the digest transit in the gastric tract and therefore the final amount of FGCs in faeces (Schwarzenberger *et al.*, 1996; Wasser *et al.*, 2000; Morrow *et al.*, 2002). A previous publication in our study area found that *G. agilis* present seasonal diet shifts, including more fruits in their diet during the dry season and consuming more insects during the wet season (de Camargo *et al.*, 2014). Hence to control this confounding factor, the concentration of FGCs were expressed in gram of faecal sample (Morrow *et al.*, 2002). For that, all faecal pellets collected from individual *G. agilis* were dried, and crushed in a mortar, passed through a fine sieve, and 110–130 mg of dry faecal powder were weighed into glass tubes. Subsequently, 3 ml of 80% ethanol (ETOH) were added marking the level of liquid in each tube. The tubes were heated in a water boiling bath at a temperature of 90–100°C for 20 min. The tubes were refilled with ETOH to the mark to recover the initial volume, and centrifuged at 1500 rpm for 20 min. The supernatant was decanted into a second set of glass tubes and the liquid evaporated with compressed air in a bath of warm water at 36°C. The sediment was redissolved with 500 μl of 100% ETOH and stirred in a vortex for 30 min and left standing for 30 min. After this time, the tubes were centrifuged at a speed of 15 000 rpm for 20 min and then the supernatant was decanted into poly propylene tubes. Finally, the tubes were sealed and kept frozen at −20°C until analysis.

The effectiveness of the GCs extraction procedure (70%) was determined by means of the recovery percentage using a known quantity of radiolabeled steroid in every extraction step. The final assay concentration was corrected for this efficiency. All extracted faecal samples in 100% ETOH were diluted 1:4 in phosphate buffered saline (PBS; 0.05 M, pH 7.4) before the determination of cortisol levels by radioimmunoassay.

The samples were analysed by commercial cortisol radioimmunoassay (CORT-CT2) developed by Cisbio Bioassays (Parc Marcel Boiteux-BP 84175-30200 Codolet/France) using the procedure for salivary cortisol measurement. The specificity of the assay was as follows: 100% cortisol; 2.5% corticosterone; 2.2% cortisone; 1.5% 21-deoxycortisol; 1.2% 11-deoxycortisol; 1.2% prednisone and < 0.3 with all other steroids. Inter-assay variation was 4.9% of coefficient of variation (CV) (20 assays), while intra-assay variation was 6.9% CV. The analytical sensitivity of the assay was 0.04 ng/ml or 2.6 pg per loaded sample.

Serial dilutions of faecal extracts run against the Cisbio assay kit standards gave satisfactory degree of parallelism for the assay (*F*_(9,12)_0.7105; *P* = 0.6913). Assays data were analysed as described by Dudley *et al.* (1985).

### Parasite analysis

Duplicates of the faecal samples of *G. agilis* used for evaluating cortisol metabolites were analysed to detect the presence of gastrointestinal parasites. Samples were diluted in the laboratory into a very dense sodium chloride solution to allow flotation of eggs and oocysts. When detected, eggs and oocysts were counted under a light microscope (Leica DM500, magnification 100×), and their abundance estimated per gram of faeces following the McMaster technique (Gordon and Whitlock, 1939). Only faecal samples weighing more than 0.3 g were quantitatively analysed, while samples weighing <0.3 g were analysed qualitatively for presence of parasites. The qualitative analysis was carried out by colouring several drops of the diluted faecal sample with lugol, followed by observation of samples under a light microscope (Leica DM500, magnification 400).

Nematodes and cestodes were identified to the minimum taxonomic level possible based on characteristics of eggs and previous studies with the parasites of the *G. agilis* populations. *Eimeria* spp. was identified after sporulation of oocysts present in the faeces, which were observed under a light microscope (Leica DM500, magnification 400). To sporulate oocysts, faecal samples were placed into 2.5% aqueous potassium dichromate solution (K_2_Cr_2_O_7_), then placed in a Petri dish inside a thermostatically controlled incubator at 27°C for ~5 days. Earlier studies pointed out that *Eimeria* spp. was the most prevalent and abundant gastrointestinal parasite infecting *G. agilis* individuals (Strona *et al.*, 2015), hence our analysis covers the association between *Eimeria* spp. infection and cortisol levels of *G. agilis* individuals in the population.

### Data analysis

To determine differences in faecal cortisol metabolite (FGC) levels between sample times (0, 24 and 48 h) during the capture stress challenge tests in *G. agilis,* we ran a Kruskal–Wallis test followed by Dunn’s multiple comparison test to compare differences between sum ranks with Graph Pad Prism (Graph Pad software, Inc., San Diego, CA.).

To test the effects of individual and environmental factors on cortisol metabolite levels in *G. agilis,* we used generalised linear mixed-effects models (GLMM), which handles non-linear data avoiding transformations through the use of a link function, and also non-linear effects between dependent and independent variables. A set of different models were fitted using negative binomial distribution and log-link function for cortisol values in *G. agilis*, and we included sex, reproductive status (reproductive x non-reproductive) and climatic season (dry × wet season) as categorical, fixed effects, while body mass was included as a covariate. Random effects were incorporated, due to repeated samples from the same individuals. Hence, specific effects of covariates and factors (fixed effects) were tested while accounting for the variance explained by potentially confounding variables, which are included as ‘random variables’.

To test the effects of cortisol metabolite levels on abundance of parasites (*Eimeria* spp.) in *G. agilis* individuals, we also used GLMM with a negative binomial distribution and a log-link function. A series of models were fitted to test the effects of cortisol metabolite levels on *Eimeria* spp. abundance in *G*. *agilis*. Models included cortisol metabolite level as a covariate, and the interaction between cortisol levels and categorical predictors, such as sex, climatic season and reproductive status, which were described influencing *Eimeria* spp. infection in *G*. *agilis* in a previous study (Strona *et al.,* 2015). We did not fitted models including the separate effects of the categorical predictors mentioned above, since their role were already tested in a previous study with a larger data set (see Strona *et al.,* 2015).

Analysis were carried out in R 3.4.3, using the statistical package glmmTMB. This package estimates GLMM parameters (fixed-effects and random-effects parameters, such as effects of each variable, differences among treatments, standard deviation of random effects and all interactions) by Laplace approximation, which reduces bias in estimation and is considered more accurate (Bolker *et al.* 2008). Wald statistics and its associated *P*-value were used to test the significance of regression coefficients, by accepting a significance level of *P* < 0.05. Model selection was based on Akaike information criterion corrected (AICc) for small samples, hence models with lowest AICc values (difference lower than 2 from other models) were selected as the most parsimonious.

The association between parasite infection (presence/absence of *Eimeria* spp. in *G. agilis* individuals) and cortisol levels was evaluated by testing whether individuals presenting *Eimeria* spp. (coded as 1) had higher cortisol metabolite levels in their faeces than individuals were we failed to find *Eimeria* spp. (coded as 0), through a logistic regression performed in Statistica 8.0 (Statsoft).

## Results

### Capture stress challenge experiment

**Figure 1:**
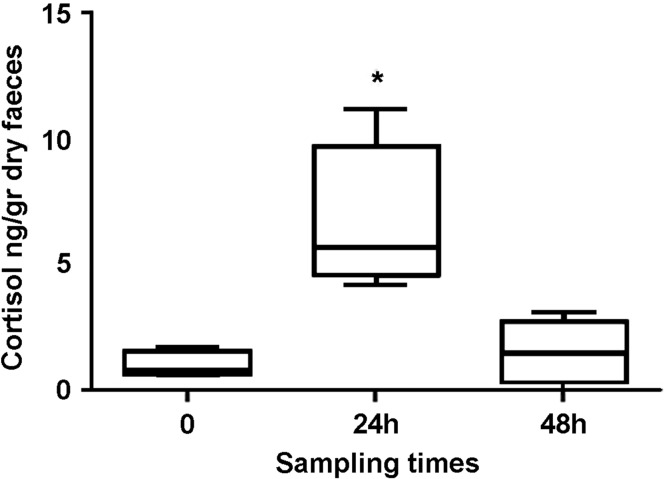
Faecal cortisol metabolites in response to 48 h capture stress in *Gracilinanus agilis* individuals (*n* = 4). *P < 0.05 (Kruskal–Wallis test followed by Dunn’s multiple comparisons test).

*Gracilinanus agilis* individuals presented mean cortisol metabolite levels of 0.9 ng/g dry faeces (SD = 0.53) before the capture challenge (time 0). Differences in cortisol metabolite levels were found between the sampling times (*H* = 7.67, *P* < 0.05), although differences were restricted to the comparison between time 0 and 24 h after (see Fig. 1). Cortisol metabolite levels increased significantly after the animals were kept in a cage for 24 h (6.66 ± 3.069 ng/g dry faeces; *P* < 0.05), however no differences were found 48 h later (1.49 ± 1.28 ng/g dry faeces; *P* > 0.05) when comparing to time 0.

### Assessment of the effect of environmental and individual factors in the levels of metabolites of cortisol in *Gracilinanus agilis* faecal samples

**Figure 2:**
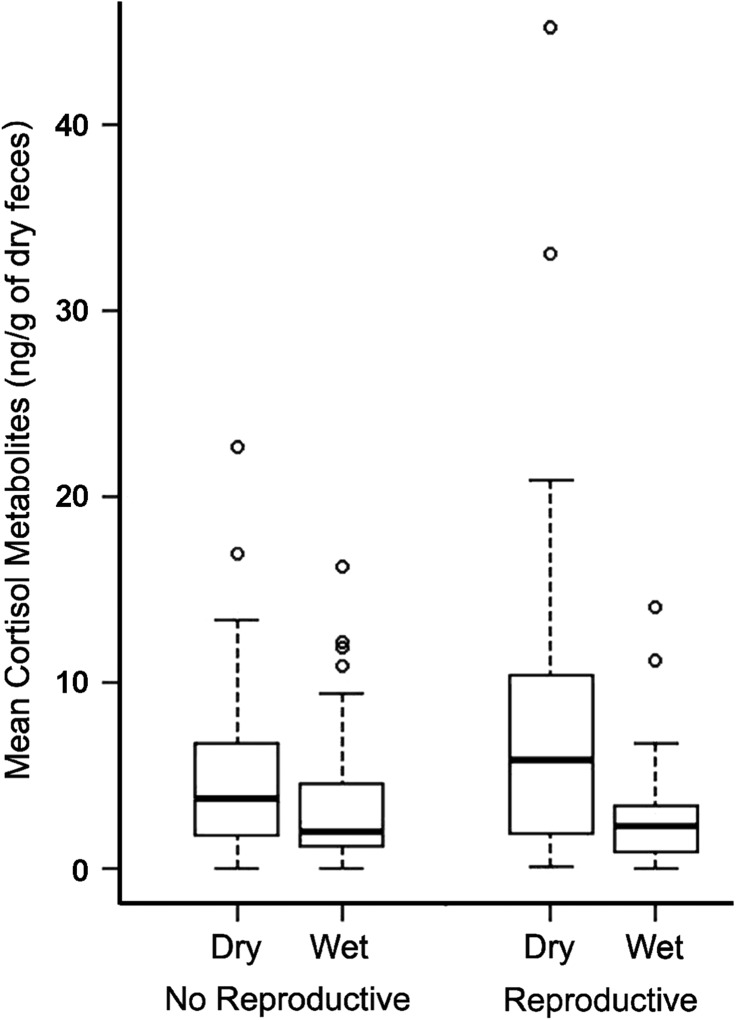
Comparison of faecal cortisol metabolites (FGC) of non-reproductive and reproductive *G. agilis* individuals during dry and wet season. Values represent Mean ± SD, *P < 0.05.

Cortisol metabolite levels in the faeces of *G. agilis* ranged from 0.0003 to 75.410 ng/g dry faeces, with a large variation among samples (SD = 8.25). Model selection showed that sex, season, reproductive status and body mass affected cortisol metabolite levels in *G. agilis*(Table 1). In the best model, FGC levels were higher during the dry season (mean and SD = 6.69 ± 6.83 ng/g dry faeces) than in the wet season (3.77 ± 3.85 ng/g dry faeces) and in reproductive (6.21 ± 6.83 ng/g dry faeces) than non-reproductive individuals (4.52 ± 4.78 ng/g dry faeces) (*X*^2^ = 36.57, *P* = 0.0001) (Fig. 2). Although body mass was included in the best model, we failed to find any statistically significant effect of this variable on FGC levels in *G. agilis* (*t*= 1.56, *P* = 0.11). On the other hand, the inclusion of body mass in this model seems to be an artefact, due to the increased body mass of reproductive individuals during the early dry season (see Lopes and Leiner, 2015), which corresponded to the period of increased FGC levels in *G. agilis*. FGC

**Table 1:** Summary of GLMM results, evaluating the negative binomial distribution models responsible for explaining FGC levels in *G. agilis* individuals at Estação Ecológica do Panga, from April 2013 to March 2015. Selected models are marked in bold

Model	AICc	AICc difference	DF
**Reproductive status + season + body mass**	1117.41	0	6
Reproductive status + season + sex + body mass	1119.45	2.04	7
Season + body mass	1126.61	9.20	5
Reproductive status + season	1126.82	9.41	5
Season	1127.47	10.06	4
Sex + season + reproductive status	1128.32	10.91	6
Sex + season + body mass	1128.33	11.92	6
Sex + season	1128.83	11.42	5
Reproductive status + body mass	1128.95	11.54	5
Reproductive status + body mass+season	1130.52	13.11	6
Season × reproductive status	1131.58	14.17	6
Season × body mass	1134.99	17.58	6
Sex × season	1137.64	20.23	6
Reproductive status × body mass	1138.88	21.47	6
Reproductive status + sex	1141.60	24.19	5
Reproductive status	1147.90	30.49	4
Sex	1149.88	32.47	4
Sex × reproductive status	1150.46	33.05	6
Sex + body mass	1151.30	33.89	5
Null model	1155.22	37.81	3
Sex × body mass	1155.29	37.88	6
Body mass	1156.40	39.01	4

Regarding parasitism, although the model including FGC levels and the interaction between FGC levels and reproductive status were selected as plausible models explaining *Eimeria* spp. abundance in *G. agilis* (see Table 2), in both models we failed to find any significant effect of cortisol metabolite levels on *Eimeria* spp. abundance in *G. agilis* (model including only FGC—*t* = 1.25, *P* = 0.21; model including the interaction between FGC and reproductive status—*t* = 0.83, *P* = 0.41). Moreover, both models were not statistically different from the null model (*X*^2^ = 5.11, *P* = 0.16 and *X*^2^ = 0.91, *P* = 0.34), suggesting that these variables lack a explanatory role on *Eimeria* spp. abundance fluctuations. However, the logistic regression shows that high levels of cortisol are associated with a higher probability of finding *Eimeira* coccidia in *G. agilis* individuals (Chi-square = 6.09, *P* = 0.01). It also demonstrates that the effect is restricted to the dry season (Chi-square (*n*) = 7.15, *P* = 0.007), rather than to the wet season (Chi-square (*n*) = 1.57, *P* = 0.21).

**Table 2:** Summary of GLMM results, evaluating the role of FGC levels and the interaction between FGC levels and categorical predictors on *Eimeria* spp. abundance in *G. agilis* individuals at Estação Ecológica do Panga, from April 2013 to March 2015. Selected models are marked in bold

Model	AICc	AICc difference	DF
**Null model**	2338.425	0	3
**FGC levels**	2339.597	1.172	4
**FGC levels × reproductive status**	2339.613	1.188	6
FGC levels × sex	2341.968	3.543	6
FGC levels × season	2343.743	5.318	6

## Discussion

Multidisciplinary approaches have had increased interest to better understand the interactions between an organism and its environment (Wingfield *et al.*, 2008). The way that an organism copes with the environment cannot be tackled from only one approach; hence, ecological, behavioural and evolutionary responses must be evaluated to better understand how the physiological control mechanisms determine optimal responses to selection processes like parasitism. To our knowledge, this is the first study that integrates environmental changes, hormone responses and parasite loads in an American marsupial, in both empirical and experimental approaches.

In recent years, there has been increasing interest in the use of non-invasive techniques to assess the stress response in wild animals and its association with different strategies to cope with environmental and anthropogenic stressors. While non-invasive techniques have several advantages over invasive techniques, there is evidence that the excretion pathway and concentrations of the hormones differ between species (Wasser *et al.,* 2000; Touma and Palme, 2005). Therefore, a physiological validation is required every time stress responses are assessed in a new species. While the pharmacologic challenge is the most accepted physiological validation for adrenal activity, there is evidence that the exposure to an unfamiliar stressor like captivity or restriction can exert an effect in the adrenal activity that is comparable to the administration of a synthetic ACTH (Harper and Austad, 2000; Narayan *et al.*, 2012; Hernández *et al.*, 2016). Despite the small number of animals used during the field capture stress challenge, we found that after 24 h of captivity, the levels of cortisol metabolites in faecal samples (FGC) increases. Our findings are similar to previous stress challenge experiments in red-back voles (*Clethrionomys gapperi*) which demonstrate in a small number of animals (n5) that the exposure of 8 h into a new caging conditions, was enough to increase FGC (Harper and Austad, 2000). Therefore, we can assume that the FGC measured in *G. agilis* faecal samples are reflex of adrenal activity.

The assessment of the effect of environmental and individual factors in the levels of metabolites of cortisol in *G. agilis* faecal samples found that dry season has a strong influence on the FGC in the studied population. There is evidence that the adrenal activity associated with a stress response increases during dry seasons in wild animal populations, due to food and water shortage that act as stressors, elevating GCs levels in wild animals (Rogovin *et al.*, 2008; Davies *et al.*, 2013; Cizauskas *et al.*, 2015). Previous research with the studied *G. agilis* population found a reduction in food availability during the mid-dry season (de Camargo *et al.*, 2014; Lopes and Leiner, 2015), as expected in cerrado and savannah habitats. In fact, other species inhabiting savannah habitats exhibit similar changes in their GC levels associated to dry season. For example, FMC concentrations of African elephants were inversely correlated with rainfall, as well as with food and water availability (Foley *et al.*, 2001). Moreover, a study in wild African ungulates found a strong seasonal effect in faecal glucocorticoid metabolites (FGM), which increase during dry season (Cizauskas *et al.*, 2015). Similar results have been found in koalas (Davies *et al.*, 2013) and Neotropical monkeys, like spider monkeys (*Ateles geoffroyi yucatanensis*) (Rangel-Negrín *et al.*, 2009) and muriquis (*Brachyteles aracnoides*) (Strier *et al.*, 1999). The consistencies in the reports from different wildlife populations, from different areas demonstrate that dry seasons impose a strong fitness challenge in animal populations.

Season also had a strong influence in the association of FGC and the other variables. For example, body mass and reproductive status by themselves did not have an effect in FGC in *G. agilis*, however, when season was included in the model an effect of both factors were observed. *G. agilis* individuals experience an increase in body mass during the breeding season, which starts in July (dry season), although females reach higher body mass during the lactation period (wet season) (Lopes and Leiner, 2015). Hence, the positive association between cortisol metabolite levels and body mass is the result of the effect of season and reproduction on body mass and FGC levels of *G. agilis*. Regarding reproduction, several studies have already demonstrated higher FGM levels due to increased costs of reproduction among vertebrates (Romero, 2002; Moore and Jessop, 2003; Naylor *et al.,* 2008). Within *G. agilis*, it seems that the costs of reproductive activity elicit a stress response, increasing FGC levels only when associated to unfavourable conditions that occur during the dry season. In fact, the mid-late dry season corresponds to the mating period, when there seems to be a strong competition for mates among males, which is supported by the presence of signs of aggression among them (i.e. wounds) and a male-biased sex ratio in the studied population (Lopes and Leiner, 2015). Moreover, within females, this period corresponds to gestation and lactation of the first litter, while a second litter is weaned under favourable conditions during the wet season (Lopes and Leiner, 2015). Our results are mirrored to a study in wild African ungulates, in which peak of FGM are driven by dry season as well as reproductive season (Cizauskas *et al.*, 2015). Hence, our results support the interplay between stress response, reproductive status and weather.

**Figure 3:**
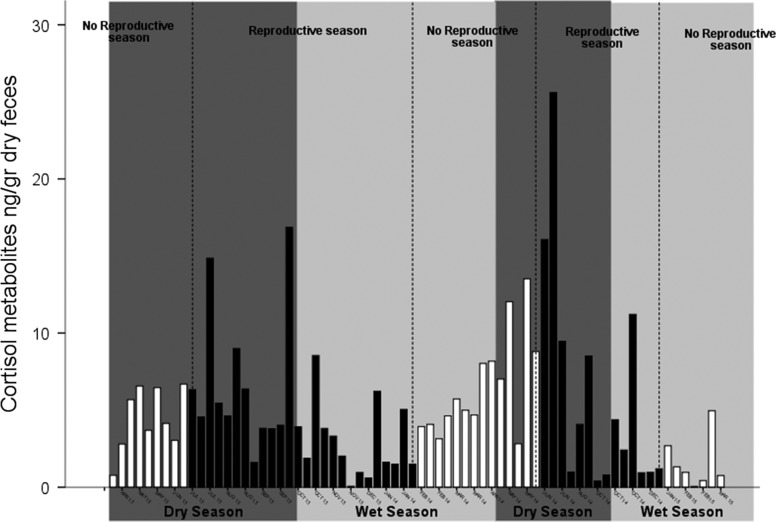
Monthly fluctuations in faecal cortisol metabolites (FGC) in *Gracilinanus agilis* individuals captured between April 2013 and March 2015 at Estação Ecologica do Panga, MG, Brazil. Bars represent individual FGC levels in different sampling points.

In the studied population, FGCs varied seasonally, with increased levels during the dry, reproductive season (see Fig. 3). Several species of vertebrates modulate GCs concentrations seasonally, with elevated levels during adverse conditions and/or energetically costly periods of the year. While there is evidence of the existence of seasonality in GCs in several wild animal populations, the biological and ecological function of these phenomena is still in discussion. To explain this phenomenon some researchers have proposed that GCs act as a buffer increasing or reducing physiological and behavioural responses (Sapolsky *et al.,* 2000; Romero, 2002; McEwen and Wingfield, 2003; Denver, 2009). However, there is evidence that its final effect on the life histories is influenced by environmental cues that are registered as energy or resources access (Denver, 2009). Moreover, GCs are a good predictor of future survival. For example, high levels of GCs due to stressful conditions are associated with mortality on the next season (Romero and Wikelski, 2001; Rogovin *et al.*, 2003), low reproductive success (Vitousek and Romero, 2013) as well as higher probability of future disease presentation (Cizauskas *et al.*, 2015; Oppliger *et al.*, 1998; Pedersen and Greives, 2008).

This final point was demonstrated in our study. We found that an increase in FGC levels among *G. agilis* during the dry season is associated with a rise in the probability of being infected by *Eimeria* spp., which was the most common and abundant parasite infecting *G. agilis* individual in the study site (Strona *et al.* 2015). *Eimeria* spp. is an opportunistic parasite so a compromised immune competence, and behaviours that increase the exposure to the parasite in an highly infected environment, are risk conditions that can lead to coccidian outbreak (Chartier and Paraud, 2012). The highest levels of FGC among *G. agilis* individuals previous to the coccidian outbreak that occurred in the wet/breeding season in the studied population (Strona *et al.* 2015) suggests an immunosuppressive effect of the GCs, which have been widely documented in several vertebrate species (Bourgeon and Raclot, 2006; Roberts *et al.*, 2007). Moreover, *G. agilis* spacing patterns during the breeding season seems to contribute to the increased exposure of both sexes to parasite infection. Usually, male marsupials, including *G. agilis*, extend their home range during the reproductive period to increase its access to mates (Loretto and Vieira, 2005). On the other hand, females present smaller home ranges and exhibit territorial behaviour (Lopes, 2014), which might reduce its chances either to be exposed to parasites or kept high parasite loads (Strona *et al.,* 2015). However, during breeding season there is a home range overlap between females and males (Lopes, 2014), which might increase the probability of infection either from males carrying high parasite loads, or from females inhabiting focus of high parasites loads (Strona *et al.,* 2015).

Finally, while there is evidence of the immunosuppressive effect of high levels of GCs and space use patterns for both sexes which might contribute to high loads of *Eimeria* spp. in *G. agilis*, we cannot exclude the possibility of environmental stress due to food restriction. Individuals carrying *Eimeria* spp. occysts during the dry season might then present higher FGC levels than individuals lacking *Eimeria* spp. oocysts, due to the synergistic negative impacts of parasite infection and food shortage in *G. agilis* hosts. Pedersen and Greives (2008) found that the increase in FGC levels among infected white-footed mice was restricted to the low food availability season. Irrespectively, we suggest that a rise in GCs during the dry season may contribute to the observed increased parasite loads within *G. agilis* males and females during the wet season (Strona *et al.,* 2015).

Our results suggest that the chronic activation of the adrenal activity during the dry season seems to contribute to population crashes after reproductive season in G. *agilis*, associated with high parasite loads. Hence, our finding supports the corticosteroid-fitness hypothesis, which predicts that increased GCs as a response to stressors usually results in decreased fitness of individuals, translated into low future survival and reproductive success, and higher parasite infection (Bonier *et al.*, 2009). There is evidence of stress-related die-off in semelparous dasyurid marsupials after handicapped life-history periods like reproductive season (Bradley *et al.*, 1980; Dickman and Braithwaite, 1992; Naylor *et al.,* 2008). Male die-off has also been observed with semelparous didelphids (Leiner *et al.,* 2008), and other mammals with short life-spans and early reproduction (Delehanty and Boonstra, 2011). However, previous studies failed to find a role of GCs in the occurrence of a fast life-history in *Didelphis virginiana* (Woods and Hellgren, 2003) and artic ground squirrels (Delehanty and Boonstra, 2011), probably due to maintenance of CBG binding capacity which eliminates the cumulative effects of free GCs levels. Hence, to test the role of a rise in GCs in *G. agilis* on the occurrence of male die-off, CBG binding capacity and free GCs levels need to be evaluated in future studies.

## Conclusions

The present study demonstrates that FGCs are a reliable indicator of adrenal activity in *G. agilis*, which is associated with an increase of the stress response during a food scarcity period, like the dry season. In the studied population, FGCs varied seasonally, and the increased levels during the dry season seems to decrease future survival and increase the probability of disease presentation, demonstrated by coccidian outbreaks during the wet, reproductive season in *G. agilis* individuals and male post-mating die-off in this species. We also provide evidence that the positive association between FGCs and body mass is the result of the effect of season and reproduction in both variables.

## References

[coy021C1] Baladrón AV, Malizia AI, Bó MS, Liébana MS, Bechard MJ (2013) Population dynamics of the southern short-tailed opossum (*Monodelphis dimidiata*) in the pampas of Argentina. Aust J Zool 60: 238–245.

[coy021C501] Bolker BM, Brooks ME, Clark CJ, Geange SW, Pulsen JR, Stevens MHH, White JS (2008) Generalized linear mixed models: a practical guide for ecology and evolution. Trends Ecol Evol 24: 127–135. 10.1016/j.tree.2008.10.008 19185386

[coy021C2] Bonier F, Martin PR, Moore IT, Wingfield JC (2009) Do baseline glucocorticoids predict fitness? Trends Ecol Evol 24: 634–642. 19679371 10.1016/j.tree.2009.04.013

[coy021C3] Bourgeon S, Raclot T (2006) Corticosterone selectively decreases humoral immunity in female eiders during incubation. J Exp Biol 209: 4957–4965. 17142684 10.1242/jeb.02610

[coy021C4] Bradley AJ (2003) Stress, hormones and mortality in small carnivorous marsupials In Jones M, Dickman CR, Archer M, eds Predators with Pouches—*The Biology of Carnivorous Marsupials*. CSIRO Publishing, Victoria, Australia, pp 255–267.

[coy021C5] Bradley AJ, McDonald IR, Lee AK (1980) Stress and mortality in a small marsupial (*Antechinus stuartii,*Macleay). Gen Comp Endocrinol 40: 188–200. 6245013 10.1016/0016-6480(80)90122-7

[coy021C6] Brownie A (1992) The metabolism of adrenal cortical steroids In James VHT, ed The Adrenal Gland, Ed 2 Raven Press Ltd, New York, pp 209–224.

[coy021C7] Chartier C, Paraud C (2012) Coccidiosis due to eimeria in sheep and goats, a review. Small Rumin Res 103: 84–92. 10.1016/j.smallrumres.2011.10.023 PMC7135340 32288206

[coy021C8] Cizauskas CA, Turner WC, Pitts N, Getz WM (2015) Seasonal patterns of hormones, macroparasites, and microparasites in wild African ungulates: the interplay among stress, reproduction, and disease. PLoS One 10: e0120800. 25875647 10.1371/journal.pone.0120800 PMC4398380

[coy021C9] Cockburn A (1997) Living slow and dying young: senescence in marsupials In Saunders N, Hinds L, eds Marsupial Biology: Recent Research, New Perspectives. University of New South Wales Press, Sydney. University of New South Wales Press, Sydney, pp 163–171.

[coy021C10] Davies NA, Gramotnev G, McAlpine C, Seabrook L, Baxter G, Lunney D, Rhodes JR, Bradley A (2013) Physiological stress in koala populations near the arid edge of their distribution. PLoS One 8: e79136. 24265749 10.1371/journal.pone.0079136 PMC3827162

[coy021C11] de Camargo NF, Ribeiro JF, de Camargo AJA, Vieira EM (2014) Diet of the gracile mouse opossum *Gracilinanus agilis* (Didelphimorphia: Didelphidae) in a neotropical savanna: intraspecific variation and resource selection. Acta Theriol 59: 183–191.

[coy021C12] Delehanty B, Boonstra R (2011) Coping with intense reproductive aggression in male arctic ground squirrels: the stress axis and its signature tell divergent stories. Physiol Biochem Zool 84: 417–428. 21743255 10.1086/660809

[coy021C13] Denver RJ (2009) Structural and functional evolution of vertebrate neuroendocrine stress systems. Ann N Y Acad Sci 1163: 1–16. 19456324 10.1111/j.1749-6632.2009.04433.x

[coy021C14] Dickman CR, Braithwaite RW (1992) Postmating mortality of males in the dasyurid marsupials, Dasyurus and Parantechinus. J Mammal 73: 143–147.

[coy021C15] Dloniak SM, French JA, Place NJ, Weldele ML, Glickman SE, Holekamp KE (2004) Non-invasive monitoring of fecal androgens in spotted hyenas (*Crocuta crocuta*). Gen Comp Endocrinol 135: 51–61. 14644644 10.1016/j.ygcen.2003.08.011

[coy021C503] Dudley RA, Edwards P, Ekins RP, Finney DJ, McKensey GI, Raab GM, Robard D, Rodgers RP (1985) Guidelines for immunoassay data processing. Clin Chem 31: 1264–1271. 3893796

[coy021C16] Foley CAH, Papageorge S, Wasser SK (2001) Noninvasive stress and reproductive measures of social and ecological pressures in free-ranging african elephantsestrés no invasivo y medidas reproductivas de presiones sociales y ecológicas en elefantes africanos libres. Conserv Biol 15: 1134–1142.

[coy021C504] Gordon HM, Whitlock HV (1939) A new technique for counting nematode eggs in sheep faeces. J Sci Ind Res 12: 50–52.

[coy021C17] Graham LH, Brown JL (1996) Cortisol metabolism in the domestic cat and implications for noninvasive monitoring of adrenocortical function in endangered felids. Zoo Biol 15: 71–82.

[coy021C18] Harper JM, Austad SN (2000) Fecal glucocorticoids: a noninvasive method of measuring adrenal activity in wild and captive rodents. Physiol Biochem Zool 73: 12–22. 10685902 10.1086/316721

[coy021C19] Hernández SE, Sernia C, Bradley AJ (2016) Adrenocortical function in cane toads from different environments. Comp Biochem Physiol A 195: 65–72. 10.1016/j.cbpa.2016.02.001 26877241

[coy021C20] Hing S, Narayan E, Thompson RCA, Godfrey S (2014) A review of factors influencing the stress response in Australian marsupials. Conserv Physiol 2: cou027–cou027. 27293648 10.1093/conphys/cou027 PMC4732483

[coy021C21] Lafferty KD, Holt RD (2003) How should environmental stress affect the population dynamics of disease? Ecol Lett 6: 654–664.

[coy021C22] Leiner NO, Setz EZF, Silva WR (2008) Semelparity and factors affecting the reproductive activity of the Brazilian slender opossum (*Marmosops paulensis*) in southeastern Brazil. J Mammal 89: 153–158.

[coy021C23] Lopes GP (2014) Reproductive strategy and spatial organization of a Gracilinanus agilis (Didelphimorphia: Didelphidae) population at Estação Ecológica do Panga, em Uberlândia/MG. Master, Universidade Federal de Uberlândia, Brazil.

[coy021C24] Lopes GP, Leiner NO (2015) Semelparity in a population of *Gracilinanus agilis* (Didelphimorphia: Didelphidae) inhabiting the Brazilian cerrado. Mamm Biol—Z für Säugetierkunde 80: 1–6.

[coy021C25] Loretto D, Vieira MV (2005) The effects of reproductive and climatic seasons on movements in the black-eared opossum (*Didelphis aurita* Wied-Neuwied, 1826). J Mammal 86: 287–293.

[coy021C26] Madliger CL, Love OP (2014) The need for a predictive, context-dependent approach to the application of stress hormones in conservation. Conserv Biol 28: 283–287. 24283988 10.1111/cobi.12185

[coy021C27] Martin LB (2009) Stress and immunity in wild vertebrates: timing is everything. Gen Comp Endocrinol 163: 70–76. 19318107 10.1016/j.ygcen.2009.03.008

[coy021C28] McEwen BS, Wingfield JC (2003) The concept of allostasis in biology and biomedicine. Horm Behavior 43: 2–15. 10.1016/s0018-506x(02)00024-7 12614627

[coy021C29] Meikle WA (1989) Secretion and metabolism of the corticosteroids and adrenal function and testing In DeGroot JL, ed Endocrinology, Ed 2 Vol. 2 W. B. Saunders Company, London, pp 1610–1632.

[coy021C30] Moore IT, Jessop TS (2003) Stress, reproduction, and adrenocortical modulation in amphibians and reptiles. Horm Behavior 43: 39–47. 10.1016/s0018-506x(02)00038-7 12614633

[coy021C31] Morrow CJ, Kolver ES, Verkerk GA, Matthews LR (2002) Fecal glucocorticoid metabolites as a measure of adrenal activity in dairy cattle. Gen Comp Endocrinol 126: 229–241. 12030779 10.1006/gcen.2002.7797

[coy021C32] Möstl E, Palme R (2002) Hormones as indicators of stress. Domest Animal Endocrinol 23: 67–74. 10.1016/s0739-7240(02)00146-7 12142227

[coy021C33] Narayan EJ, Cockrem JF, Hero J-M (2012) Effects of temperature on urinary corticosterone metabolite responses to short-term capture and handling stress in the cane toad (*Rhinella marina*). Gen Comp Endocrinol 178: 301–305. 22728158 10.1016/j.ygcen.2012.06.014

[coy021C34] Naylor R, Richardson SJ, McAllan BM (2008) Boom and bust: a review of the physiology of the marsupial genus *Antechinus*. J Comp Physiol B 178: 545–562. 18210128 10.1007/s00360-007-0250-8

[coy021C505] Oliveira-Filho AT, Ratter JA (2002) Vegetation physiognomies and woody flora of the cerrado biome In Oliveira PS, Marquis RJ, eds The Cerrado of Brazil. Columbia University Press, New York, pp 91–120.

[coy021C35] Oppliger A, Clobert J, Lecomte J, Lorenzon P, Boudjemadi K, John-Alder HB (1998) Environmental stress increases the prevalence and intensity of blood parasite infection in the common lizard *Lacerta vivipara*. Ecol Lett 1: 129–138.

[coy021C36] Palme R, Robia C, Baumgartner W, Mostl E (2000) Transport stress in cattle as reflected by an increase in faecal cortisol metabolite concentrations. Vet Rec 146: 108–109. 10682697 10.1136/vr.146.4.108

[coy021C37] Pedersen AB, Greives TJ (2008) The interaction of parasites and resources cause crashes in a wild mouse population. J Animal Ecol 77: 370–377. 10.1111/j.1365-2656.2007.01321.x 18028357

[coy021C38] Rangel-Negrín A, Alfaro JL, Valdez RA, Romano MC, Serio-Silva JC (2009) Stress in Yucatan spider monkeys: effects of environmental conditions on fecal cortisol levels in wild and captive populations. Animal Conserv 12: 496–502.

[coy021C39] Raouf SA, Smith LC, Brown MB, Wingfield JC, Brown CR (2006) Glucocorticoid hormone levels increase with group size and parasite load in cliff swallows. Anim Behav 71: 39–48.

[coy021C40] Roberts ML, Buchanan KL, Hasselquist D, Evans MR (2007) Effects of testosterone and corticosterone on immunocompetence in the zebra finch. Horm Behav 51: 126–134. 17049519 10.1016/j.yhbeh.2006.09.004

[coy021C41] Rogovin K, Randall JA, Kolosova I, Moshkin M (2003) Social correlates of stress in adult males of the great gerbil, *Rhombomys opimus*, in years of high and low population densities. Horm Behavior 43: 132–139. 10.1016/s0018-506x(02)00028-4 12614643

[coy021C42] Rogovin KA, Randall JA, Kolosova IE, Moshkin MP (2008) Long-term dynamics of fecal corticosterone in male great gerbils (*Rhombomys opimus licht.*): effects of environment and social demography. Physiol Biochem Zool 81: 612–626. 18781838 10.1086/588757

[coy021C43] Romano MC, Rodas AZ, Valdez RA, Hernández SE, Galindo F, Canales D, Brousset DM (2010) Stress in wildlife species: noninvasive monitoring of glucocorticoids. Neuroimmunomodulation 17: 209–212. 20134205 10.1159/000258726

[coy021C44] Romero LM (2002) Seasonal changes in plasma glucocorticoid concentrations in free-living vertebrates. Gen Comp Endocrinol 128: 1–24. 12270784 10.1016/s0016-6480(02)00064-3

[coy021C45] Romero LM (2004) Physiological stress in ecology: lessons from biomedical research. Trends Ecol Evol 19: 249–255. 16701264 10.1016/j.tree.2004.03.008

[coy021C46] Romero LM, Wikelski M (2001) Corticosterone levels predict survival probabilities of Galapagos marine iguanas during EL Nino events. Proc Natl Acad Sci U S A 98: 7366–7370. 11416210 10.1073/pnas.131091498 PMC34674

[coy021C47] Sapolsky RM, Romero LM, Munck AU (2000) How do glucocorticoids influence stress responses? Integrating permissive, suppressive, stimulatory, and preparative actions. Endocr Rev 21: 55–89. 10696570 10.1210/edrv.21.1.0389

[coy021C48] Schwarzenberger F, Mostl E, Palme R, Bamberg E (1996) Faecal steroid analysis for non-invasive monitoring of reproductive status in farm, wild and zoo animals. Anim Reprod Sci 42: 515–526.

[coy021C49] Strier KB, Ziegler TE, Wittwer DJ (1999) Seasonal and social correlates of fecal testosterone and cortisol levels in wild male muriquis (*Brachyteles arachnoides*). Horm Behavior 35: 125–134. 10.1006/hbeh.1998.1505 10202120

[coy021C50] Strona ALS, Levenhagem M, Leiner NO (2015) Reproductive effort and seasonality associated with male-biased parasitism in *Gracilinanus agilis* (Didelphimorphia: Didelphidae) infected by *Eimeria* spp. (Apicomplexa: Eimeriidae) in the Brazilian cerrado. Parasitology 142: 1086–1094. 25877479 10.1017/S0031182015000402

[coy021C51] Toftegaard C, Bradley A (2003) Chemical communication in dasyurid marsupials In Jones M, Dickman C, Archer M, eds. *Predators with pouches*. CSIRO Publishing, Melbourne, pp 347-357.

[coy021C52] Toftegaard CL, McMahon KL, Galloway GJ, Bradley AJ (2002) Processing of urinary pheromones in *Antechinus stuartii* (Marsupialia: Dasyuridae): functional magnetic resonance imaging of the brain. J Mammal 83: 71–80.

[coy021C53] Toftegaards CL, Moore C, Bradley AJ (1999) Chemical characterization of urinary pheromones in brown antechinus, *Antechinus stuartii*. J Chem Ecol 25: 527–535.

[coy021C54] Touma C, Palme R (2005) Measuring fecal glucocorticoid metabolites in mammals and birds: the importance of validation. Ann NY Acad Sci 1046: 54–74. 16055843 10.1196/annals.1343.006

[coy021C506] Vieria EM, de Camargo NF, Colas PF, Ribeiro JF, Cruz-Neto AP (2017) Geographic variation in daily activity patterns of a neotropical marsupial (*Gracilianus agilis*). PLoS One 12: e0168495 10.1371/journal.pone.0168495. 28052077 PMC5215639

[coy021C55] Vitousek MN, Romero LM (2013) Stress responsiveness predicts individual variation in mate selectivity. Gen Comp Endocrinol 187: 32–38. 23524274 10.1016/j.ygcen.2013.03.009

[coy021C56] Wasser SK, Hunt KE, Brown JL, Cooper K, Crockett CM, Bechert U, Millspaugh JJ, Larson S, Monfort SL (2000) A generalized fecal glucocorticoid assay for use in a diverse array of nondomestic mammalian and avian species. Gen Comp Endocrinol 120: 260–275. 11121291 10.1006/gcen.2000.7557

[coy021C57] Wingfield JC, Williams TD, Visser ME (2008) Introduction. Integration of ecology and endocrinology in avian reproduction: a new synthesis. Philos Trans R Soc B 363: 1581–1588. 10.1098/rstb.2007.0012 PMC2394566 18048300

[coy021C58] Woods HA II, Hellgren EC (2003) Seasonal changes in the physiology of male *Virginia opossums* (*Didelphis virginiana*): signs of the dasyurid semelparity syndrome? Physiol Biochem Zool 76: 406–417. 12905127 10.1086/374285

